# Fieldservers and Sensor Service Grid as Real-time Monitoring Infrastructure for Ubiquitous Sensor Networks

**DOI:** 10.3390/s90402363

**Published:** 2009-03-31

**Authors:** Kiyoshi Honda, Aadit Shrestha, Apichon Witayangkurn, Rassarin Chinnachodteeranun, Hiroshi Shimamura

**Affiliations:** 1 Remote Sensing and GIS, Asian Institute of Technology (AIT), Pathumthani, Thailand; E-Mails: aadit@ait.ac.th (A.S.); Apichon.Witayangkurn@ait.ac.th (A.W.); rassarin@ait.ac.th (R.C.); 2 Elab Experience Inc., Japan; E-Mail: h.shimamura@elab-experience.com (H.S.)

**Keywords:** Sensor Service GRID (SSG), Sensor Web Enablement (SWE), Sensor Observation Service (SOS), fieldservers, ubiquitous network

## Abstract

The fieldserver is an Internet based observation robot that can provide an outdoor solution for monitoring environmental parameters in real-time. The data from its sensors can be collected to a central server infrastructure and published on the Internet. The information from the sensor network will contribute to monitoring and modeling on various environmental issues in Asia, including agriculture, food, pollution, disaster, climate change etc. An initiative called Sensor Asia is developing an infrastructure called Sensor Service Grid (SSG), which integrates fieldservers and Web GIS to realize easy and low cost installation and operation of ubiquitous field sensor networks.

## 1. Introduction

**Figure 1.**
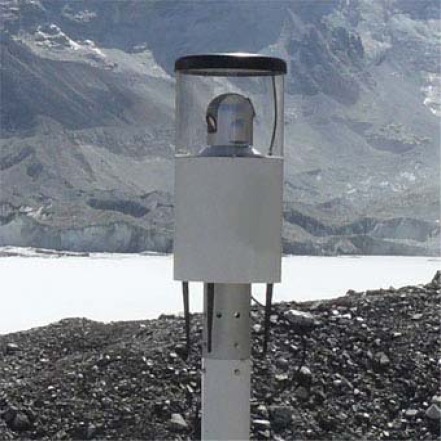
Typical Fieldserver.

The fieldserver (FS) is an Internet Field Observation Robot that consists of a set of multiple sensors, a web server, an Internet Protocol (IP) camera, as well as wireless interfaces. It is designed to provide an outdoor solution for environment monitoring. At the heart of the FS are a built-in webserver and an Analog-to-Digital converter. The analog voltage from sensors are converted and shown on webpage as table formatted data [4]. With a variety of sensors, the Fieldserver can be used for various monitoring applications such as agriculture, landslide, pollution, environment and climate monitoring just to name a few. A typical fieldserver is shown in Figure 1.

In addition to the set of sensors, the FS is equipped with an IP network camera attached to it. The camera has an Ethernet interface for communication. The camera has built-in pan (left/right) and tilt (up/down) mechanisms which can be controlled through a web browser to change the direction of the camera lens. The camera can be preset to move to different rotation and zoom positions at fixed time intervals [3].

With the wide-spread advent of communication technology, the fieldserver can now be deployed to gather data anywhere in the world where there is availability of an Internet connection. At the same time, advances in electronics and Integrated Circuits technology mean that sensors and sensing devices are getting inexpensive and more easily available. Although the fieldserver in itself is an excellent platform for data collection, tasks like sensor connection, data archiving and transfer to outside world are not easy for ordinary users. For setting up a sensor network, technical aspects such as VPN setup for global access, data archiving to a database, publishing data on the Internet, sensor configuration management etc are involved. Highly skilled engineers are required in these applications. For wide-ranging reach and use of sensor networks, a system that supports sensor ‘plug & play’ is necessary so that non-technical people can also implement their own sensor systems easily and obtain data seamlessly.

An initiative called Sensor Asia has started to fill this need with the aim of providing truly ubiquitous capabilities to sensor networks. Under this initiative, field-side agent boxes based on Sensor Observation Service (SOS), called SOS Stations, have been developed. The use of SOS Stations with facilities of the Sensor Service Grid (SSG), under Sensor Asia initiative, can simplify these tasks for general users.

## 2. Sensor Service Grid (SSG)

SSG is a sensor data middleware which provides users with a platform to receive data from remote field sensor networks. As it follows OpenGIS standards and specifications, other applications can be built based on the SSG. The SSG implementation has been designed to run in two parts – one at the sensor node in the field, i.e. the SOS Station, and the other at the SSG central server. The SOS Station is a combination of fieldserver with a small Linux Box which gives a high capability for storing sensor data and provides data connectivity to outside server using standardized data exchange protocols. The SOS Station is based on Sensor Observation Service (SOS) and the sensor data can be obtained in SensorML Observation and Measurement (O&M) encodings [6].

**Figure 2.**
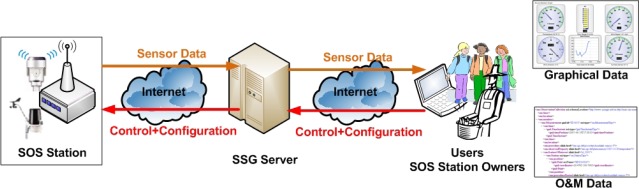
Information Flow from SOS Station to SSG to User.

The overall structure and information flow from SOS Station to SSG to users is shown in Figure 2. The SOS Station collects data from the fieldserver and other sensor systems connected to it and store it in a local database. The data is sent to the SSG server from where users can view it in graphs and maps. The data can also be obtained in standard XML O&M document. At the same time easy configuration and control of SOS Station can either be done locally or from remote locations by connecting through the SSG server. All data and configuration information are synchronized between SOS Station and the SSG server.

**Figure 3.**
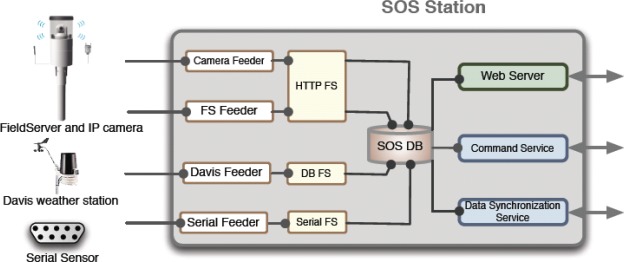
Service Layer Diagram.

The SOS Station is capable of controlling more than one fieldserver and their cameras. It also has the capability to collect data from several types of weather stations and data loggers from RS232 serial ports. As shown in Figure 3, feeder systems have been developed separately for the aforementioned devices. The feeder templates are open, supporting an open system and easy installation; any device manufacturer can utilize these templates. The webserver implemented on the SOS Station gives access to all sensor data as well as device and sensor configuration.

A command service linked to the SSG provides remote administration and configuration capabilities. SOS Station owner with proper authorization can control the system from anywhere. All data is synchronized to SSG server using messaging service. Synchronization is done not only for the sensor data, but also for sensor and device configuration.

Once deployed in the field, the SOS Station can be used to register sensors and fieldservers at the SSG central server. The sensor set can be added or changed easily with a user-friendly interface; the calibration equation and other parameters will be selected appropriately to obtain the correct sensor output, with a feel of sensor ‘plug & play’. Once registered, the SOS Station can be controlled and configured remotely from the SSG itself. Accessing to SOS Station (local access) and SSG (global access) is almost transparent to users. They can control sensor configuration and access to sensor data by accessing either of SOS Station or SSG. It overcomes the problem of local setting difficulties of remote node caused by instability of the Internet connection. SOS Station owners can access their sensor data locally or globally.

**Figure 4.**
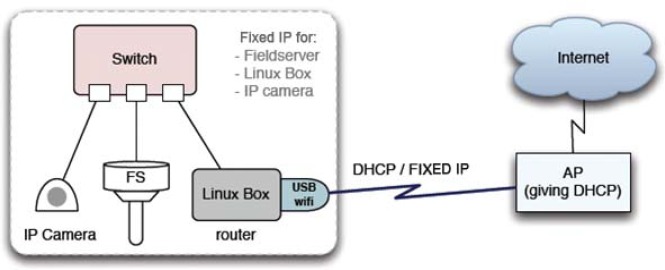
Typical Network Diagram.

The SOS Station has been made resilient to overcome firewalls and NATs, so that sensor data can be sent from any kind of Internet connection. The typical network diagram of a fieldserver deployment is shown in Figure 4. A USB WiFi interface (or an internal miniPCI WiFi card) provides access to the Internet via a wireless access point (AP). The AP may provide either fixed or DHCP IP to the Linux Box.

The primary work of the SSG implementation at the central server is the collection of data from all SOS Stations around the world, the management of all such stations, and the dissemination of information collected through the Internet. One of the main features of Sensor Asia is user-friendly data visualization.

**Figure 5.**
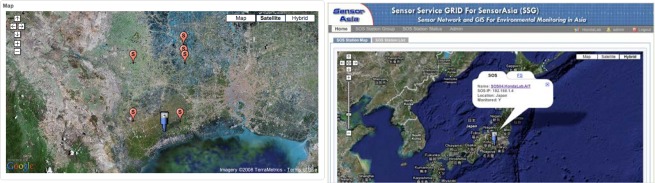
SSG showing the remote field location of SOS Station on Web GIS map.

After the SOS Station is registered at the SSG, and it starts sending data, the position of SOS Station will automatically appear on the Web GIS map, together with its list of sensors as shown in Figure 5.

**Figure 6.**
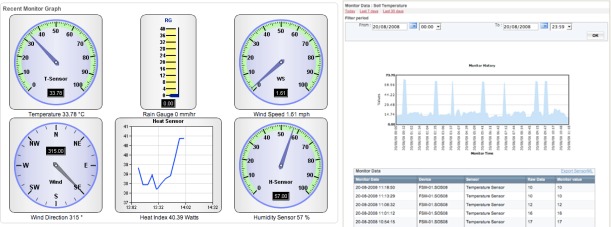
Data viewed in simple graphs and dials.

The data being received from the SOS Station can easily be viewed in real-time in the form of simple dials and graphs as show in Figure 6. Sensor configuration setup automatically creates this visual interface.

**Figure 7.**
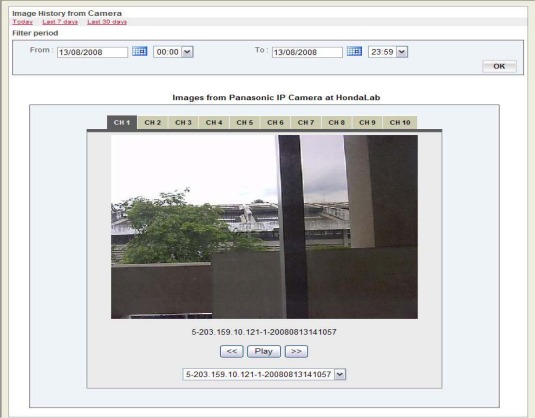
Camera interface of Sensor Asia application.

Along with the remote management of SOS Stations, the SSG application provides several levels of user management depending on authentication. This provides access to management of the remote field server from anywhere at any time with the reliability that all data is being stored at the SSG. The Sensor Asia application also provides an interface to view images from the fieldserver camera as show in Figure 7. There is a provision to provide 10 pre-sets positions for the camera, according to which the camera will rotate periodically and transfer images in all these positions.

The SSG provides a platform for ubiquitous and open sensor networks. The queries and response to and from both the SOS Stations and the SSG servers are based on standardized XML. At the core of the implementation is the OpenGIS Sensor Observation Service; sensor data are formatted in standard Observation and Measurement (O&M) encodings. A HTTP POST interface has also been implemented which supports the three core operations of SOS specification - GetCapabilities, GetObservation and DescribeSensor. Third party applications can be developed through these standard XML interfaces without having to know the sensor data collection system. Sensor manufacturers can easily make their sensors to ‘plug & play’ to the platform using the data feeder template programs of the Sensor Asia application.

## 3. Field Applications

Authors have been setting up fieldservers for various applications such as crop monitoring, landslide monitoring and glacier monitoring, as test-beds of SSG development in various parts of the Asia Pacific. Fieldservers and SOS Stations have been deployed for agricultural monitoring in Chiang Mai, Thailand, for landslide monitoring in Banjarnegara, Indonesia, and for glacier lake outburst flood (GLOF) monitoring of Imja Lake (5,000 m) in the Everest Region Himalayas, Nepal [2].

**Figure 8.**
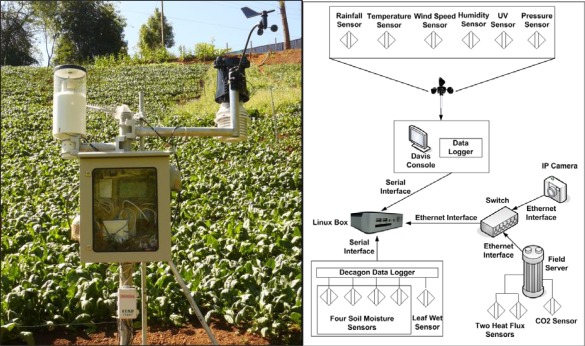
Crop monitoring field sensor network setup near Chiang Mai, Thailand.

The agricultural crop monitoring system in Chiang Mai collects various soil and weather data and sends them to SSG server at AIT. The setup consists of fieldserver with camera, soil moisture sensors (Decagon), CO_2_ sensor (SenseAir), heat flux sensors, and a weather station (Davis) as shown in Figure 8. This kind of crop monitoring system contributes to food-safety issues by improving consumer confidence about the quality of agricultural practices in the field [1]. Currently the data is being sent to a restaurant in University of Tokyo, Japan.

**Figure 9.**
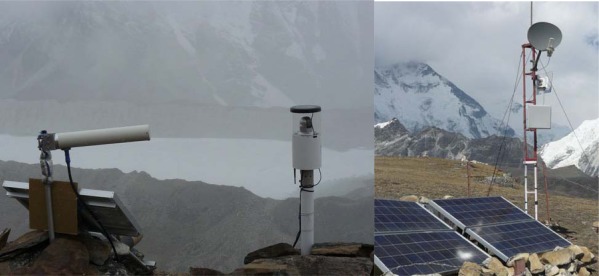
Fieldservers and wireless relay station for monitoring Imja Lake (Altitude: 5,000 m).

The Figure 9 shows one example of monitoring application in Himalayas where solar energy has been used to drive all devices and long range WiFi relay stations (up to 22 Km) have been established between fieldservers and satellite internet connection. This application is to monitor the status of the Imja Lake which has a high potential of causing a GLOF (Glacier Lake Outburst Flood) disaster [5].

## 4. Conclusions

One of the problems in setting up fieldservers and sensors in the field and their operation is that the work requires highly skilled engineers. It results in high installation cost and eventually will hinder the deployment of high density sensor networks. SSG has been developed to solve this issue. SSG supports sensor ‘plug & play’, registering sensor nodes, archiving, publishing, and visualization. These functions are important to lower the cost of installation and the make the fieldserver as off-the-shelf products for everyone. SSG supports SOS (Sensor Observation Service) as a base technology to standardize sensor information exchange within and outside of the system. The plethora of different sensors available for climatic, meteorological and agro-hydrological phenomena can be connected to the fieldservers. Sensor Asia applications based on the SSG are ideal for ubiquitous field sensor networks for different kinds of environmental monitoring.
